# Cholera

**Published:** 1855-02

**Authors:** 


CHOLERA.—Do not arrest the purging, it is eliminatory. Give turpentine and yeast as follows: yeast, a tablespoonful; oil of turpentine, a teaspoonful; cold water or barley water, two teaspoonfuls. Give this dose every second, third, or fourth hour, according to circumstances

Do not give opium. Give calomel freely, combined with camphor. Exhibit oxygen gas. This gas is easily given by shaking powdered chlorate of potash on a heated surface, and making the patient inhale the fumes. Or mix chlorate of potash with one-sixth its weight of black oxide of maganese, and throw the mixture on an iron shovel, heated to dull redness.—*Dr. Cormack.*

Lecture on Fever. By William Stokes, M. D.,— Let us now examine more closely some of the local or secondary deseases occurring in fever. In their seat, if not in their nature, these affections are observed to vary in different countries. On the continent—at least in France, and in a large portion of Germany—the frequency, and probably, the preponderance of the secondary disease of the intestines, is a matter that must be admitted. So remarkable indeed, is the predominance of the tumefaction and ulceration of the mucous glands of the intestine in France, that Andral, in the first edition of the *Clinique Médicale*, described fevers under the general head of diseases of the digestive system; and yet, Andral was no blind follower of Broussais. In Ireland, however, we do not find this remarkable preponderance of the secondary diseases of the digestive system; but, when I state this to you, I wish you to understand and adopt this principle, that all statements as to the anatomical characters of fever, as it prevails here or elsewhere, are to be accepted only so far as they apply to the prevailing epidemic. And, although it is true, that, on comparing our typhus with the French typhoid fever, this difference becomes apparent, that the existence of follicular disease of the intestine is almost the rule, and its absence the exception in the latter affection, while in the Irish typhus, this condition of the intestine is rare, you must, however, bear in mind, that in Ireland, and in our own time, we have had a great epidemic of what was certainly typhus fever, in which the condition of the intestine accurately represented that which is found to prevail on the continent. This is an important fact, and one which some of the continental writers on pathology do not seem to be aware of, or they would not be so apt to adopt artificial distinctions and erroneous opinions on the matter. In the epidemic of the years 1826, 1827, and part of 1828, disease of the mucous glands of the intestine was so frequent that its existence might be held to be the rule, and its absence the exception; and it is also true that intestinal ulcerations have been repeatedly observed in the maculated typhus of Ireland, their amount and frequency varying with the epidemic influence. Let me refer you, for proof of these statements, to Dr. Cheyne's papers in the first and second volumes of the *Dublin Hospital Reports*. Similar observations have been made elsewhere also. What, then, shall we say of that doctrine which declares that there is an essential distinction or difference marked by pathologico-anatomical characters between the continental fever and our typhus, or between the continued fever of Great Britain and fever as we have it in this country?—a difference to be expressed anatomically in this way, that in the continental or in the British continued fever there is extensive ulceration of the intestine, while in the Irish fever, this condition is wanting. Dr. Lombard, of Geneva, whose experience of fever in this country was manifestly insufficient to justify his coming to any decided opinion on the subject, holds that we have in Great Britain and Ireland two different fevers, one highly contagious, which he calls the Irish typhus, and in which the cephalic symptoms predominate to the exclusion of abdominal alterations; the other sporadic and most likely not so infectious, in which the abdominal symptoms are more predominant, so much so that the follicular disease and consequent ulceration are always present. These two fevers are in the opinion of this writer, to be found in most parts of Great Britain, but the first is most prevalent in Ireland, and in parts where the Irish come in great numbers; the other, similar to the European sporadic fever, is met with in all places, varies with the seasons, and is not necessarily produced by, or under the influence of, a contagious principle. Had Dr. Lombard been aware that ulcerations of the intestine are frequently met with in our petechial typhus; and again, that the typhoid, or at least a fever with extensive dothinenteritis, has raged epidemically for two years in this country, he would have been slow in venturing to settle the question in so decided a manner; and I have before mentioned to you, that during the prevalence of any particular epidemic in this country, we meet with cases agreeing in all their general characteristics, and having all the distinctive marks of typhus; in one set of which follicular ulceration is met with, while in another this lesion is entirely absent.

If, however, we inquire, what is the disease secondary to typhus, for the removal or modification of which we are most often called on to exert our skill in the wards of a Fever Hospital, and especially in the worst cases of petechial typhus, I would say that it is the pulmonary, rather than the gastro-intestinal complications. Certainly the secondary disease of the bronchial surface is generally the most formidable of the local affections in our Irish typhus. This is true, at least for cases which have occurred within the last ten or fifteen years. So great, indeed, is the frequency of this bronchial complication, that long before I was aware of the opinions of Rokitansky on this point, I frequently suggested in my lectures, whether the secondary bronchial disease might not be held to stand in the same relation to the Irish typhus that the follicular disease of the intestines in France bears to the fever in that country. Let me read to you a passage from Rokitansky's work, lately translated by Dr. Day for the Sydenham Society, bearing on the points before us. He is speaking of the typhus process on the mucous menbrane of the air passages:—"In primary broncho-typhus the general disease originally localises itself here, avoiding all other mucous membranes, even that of the intestine, for which in general the typhus process shows the most decided preference. The latter mucous membrane exhibits, however, in many cases a recognisable, though always subordinate and secondary developement of the follicles, in which the adjacent mesenteric glands pari cipate; and, in such cases, it is very often a difficult matter to distinguish the typhus in the above named affection of the bronchial mucous membrane. The peculiar stasis of the spleen, and of the great *cul de sac* of the stomach, the remarkable intumescence of the former, and the singular character of the blood, the typhus nature of the general disease, and especially the altered condition of the bronchial glands invariably serve, together with other symptoms, to indicate the typhus nature of the bronchial affection. The alteration occurring in the bronchial glands is of the same character as that affecting the mesenteric glands in abdominal typhus. They become swollen to the size of a pigeon's, or even of a hen's egg, are of a dark violet color, which afterwards becomes lighter, present a relaxed and friable appearance, and are infiltrated with a medullary typhus matter. Like typhus mesenteric glands, they may become the seat of a tumultuous metamorphosis, and thus either with or without perforation of the adjacent mediastinum, may give rise to pleurisy. This form is often combined with pneumo-typhus and typhus pleurisy, and is, beyond all doubt, the basis of the spotted contagious typhus, and very probably also of Irish and North American typhus, which, in the majority of cases, run their course without any intestinal affection. With us this affection is rare, and, in point of frequency, is not to be compared with abdominal typhus."

I may observe here, that by the term "tumultuous metamorphosis" is implied the occurrence of violent symptoms with suddenness and rapidity of progress. Dr. Day remarks that Rokitansky has used the term in a new sense, but it means generally, as I understand it, that a violent and reäctive inflammation occurs in the parts altered by the typhus process, which may cause great congestion, turgescence, perforation, or even gangrene. As Rokitansky does not indicate any distinctive features between the spotted contagious typhus and the Irish and North American typhus, we may safely hold them to be the same diseases.

With respect to the question of the comparative frequency and importance of the pulmonary as contrasted with the nervous symptoms in fever, as it effects the lower and upper classes in this country, my decided impression is, that taking the experience of the last twenty-five years, the secondary bronchial, or, to speak more generally, the pulmonary complications are much more frequent and dangerous in hospital than in private practice. It is not easy to explain why this should be so, but certainly we find a greater preponderance of nervous symptoms in the typhus fever, as it effects the upper classes of society, than in cases of the disease as we meet with it in hospital; while in hospital practice the nervous symptoms, though we cannot say that they are absent, seldom require any very special interference on the part of the practitioner. No doubt we meet with coma and subsultus tendinum occasionally, but that predominance of nervous symptoms in the early periods of typhus, which is so common in the upper classes of society, is but rarely seen in our wards; and if you will only reflect on the simple fact, that we so rarely have occasion to shave the head among our hospital patients, you will fully see the truth of what we say. Remember, too, how many cases we have had, in which, while all the symptoms of typhus—such as prostration, weakness of the heart, eruptions of maculæ and petechiæ, and well marked secondary diseases of the mucous membrane of the intestines—were present while the patients minds remained quite unclouded, and while no symptom occurred calling for any special measures directed to the heads. The typhus of Ireland, then, is not characterized, as Dr. Lombard has described it, by a preponderance of cephalic symptoms, at least when it occurs in that class from which he supposes the best specimens of the disease to be drawn. He is as incorrect in his statements about the predominance of cephalic symptoms as when he says that the absence of follicular ulcerations of the intestines is a distinctive mark of Irish typhus.

In the typhus fever of the upper classes in this country the nervous symptoms are generally much more aggravated and developed at an earlier period; and it may be that this preponderance of the nervous symptoms, this tendency to affections of the brain in one class, even though these affections be principally neurotic, is a cause of the comparative exemption of such cases from the secondary bronchial disease. You will find as you advance in the study of general pathology, plenty of examples in which diseases of structure or of deposition are suspended or replaced by purely nervous affections. Explain it as we will, the general proposition appears true, that the nervous symptoms, comparatively speaking, are but slightly developed in the fever of the lower classes, while those indicative of nervous disease are much more prominent; and conversely that in the upper ranks symptoms indicative of irritation of the mucous surfaces are less developed, while the nervous symptoms are severe; and this, perhaps, may throw some light upon the doctrine which has been long held by many, that the mortality of fever is greater in proportion as we ascend in the scale of society.

I have already told you that, as regards prognosis, the preponderance of the nervous symptoms in fever should cause us more apprehension than that of any other class of symptoms, and especially when this preponderance is observed at an early period of the disease. This greater susceptibility of the nervous system in the higher classes may itself be a cause why we cannot use wine with as great liberality in this class as with ordinary hospital patients. And this, again, may explain the greater fatality in private practice, inasmuch as that, in consequence of the excitement of the nervous system, and the incapability of the patients to bear stimulants largely, we are debarred from the use of that which is the great medicine for fever.

I think we may lay it down as a principle, that the more the secondary affections of fever are anatomical, the greater will be the utility of stimulants; and conversely the more they are neurotic, and especially in cases where these neurotic symptoms are more closely related to the cerebro-spinal centres, the less will be the efficacy of wine. It might be supposed, from *à priori* considerations, that the man in the higher classes of life who has been accustomed to the use of wine, would require it more and derive greater benefit from it in the course of a fever which it must be remembered is a disease of debility and prostration, than the peasant who has not been habituated to the regular use of stimuli; but I believe that the very reverse is the fact, and that the greatest triumphs of the stimulant treatment in fever are to be found when it affects the agricultural peasant whose nervous system has not been excited either by the use of stimulants or by intellectual exertion. I know that some will object to this on the ground that our Irish peasantry are habitually intemperate; but this charge of intemperance is only one of the many calumnies that have been put forth with reference to our countrymen.

The Irish peasant is not habitually intemperate; neither do his means permit nor his inclinations direct him to be so. When he does indulge, it is on special occasions; but you seldom meet in hospital a case of injury to the general health produced by a long course of intemperance in the agricultural peasant. The case is no doubt different in the class of artisans who are the inhabitants of our large cities and towns. In this class we find, that when fever attacks an individual we can neither use stimulants with the same boldness, nor reckon on their success with the same confidence as in the more sober portions of the population. In this respect the Irish are not peculiar.

To a person who is not in the habit of examining patients very closely, the frequency and importance of the bronchial disease would not appear very evident or striking as he walked round the wards of an Irish Fever Hospital. We find the patient lying in bed; he is, perhaps, in a state of stupor, but from this he is easily roused. He is covered with petechial spots, red or livid, as the case may be. There is nothing remarkable about his breathing; he is not reported to have any cough; his pulse is probably 120, or between 120 and 130, and his heart is weak. That patient, with but little hurry of breathing, not complaining of any distress, and without cough, may be, and frequently is, at that very time, almost in a very advanced stage of the bronchial disease. When you apply the stethoscope, you are surprised at the great amount of disease that it reveals, although the symptoms of this important affection are absent. One would expect, that in such a case there would at all events be lividity of the countenance from the non-arterialization of the blood; but here is one of the curious things connected with fever which it is extremely difficult to explain; that you will often see but little lividity in such cases. At all events, it is not sufficient to draw the attention of the uninformed practitioner to the condition of the patient's lung. In most patients laboring under bad typhus there is a peculiar dusky hue of the face; and this he would have whether the bronchial disease were present or not. But I have over and over again seen the most extensive bronchial disease, where every tube seemed to be half-filled, and yet little of the skin of lividity which we see in a case of asthma or if bad suffocative catarrh existed. Consider this fact for a moment, for it is full of matter for reflection. What does it tell you? It announces to you this highly important principle, that in their formation, and in their progress, the secondary diseases of fever are, as it were, silent. They occur without the usual symptoms which are observed in idiopathic inflammations. They are not idiopathic inflammations, and, therefore, they have not the symptoms of them. Let us omit no opportunity of impressing this on our minds, that these diseases are not inflammations. They form silently—they advance silently. Consider for a moment the formation of a pustule in an ordinary case of small-pox. The pustule gives no notice of its formation. You turn down the clothes, and you find the arm covered with vesicles; the patient has not complained, but you find there is the disease. So it is with respect to the bronchial disease in typhus; so it is with respect to the intestinal disease in typhus. We see the bronchial disease forming in this singular and latent manner, without excitement or suffering of the patient. We see the same in the intestine; we see the same in the heart. The softening of the heart, as you will have abundant opportunities of seeing this season, is, in its commencement and all through, a silent process. The process of change up to its maximum alteration, and the process of retrocession up to the period of recovery, considered simply, when they are not interfered with by accidental irritative inflammation, go on silently. The disease may proceed to its maximum without symptoms, and retrocede without symptoms. Now, under these circumstances, I have told you, that when you apply the stethoscope, you find singularly extensive râles. These râles may be sonorous, mucous, or crepitating; the amount of each of these characteristics varying in every case, and in different portions of the lung, in the same individual. When you use percussion, you find that there is no remarkable dullness anywhere. I have not observed, at least in the bronchial affections of typhus fever, the curious result of percussion which we see in other cases of bronchial disease. We have not observed any increase of sonoriety of the chest. This is a very remarkable fact. In a large number of cases of acute inflammatory bronchitis, you observe, that, so far from the chest being dull, it is actually clearer than natural; and this is explained by the circumstance, that there is a great accumulation of air in the air-cells of the lung,—that, in fact, every little air-cell of the lung is in a condition of extreme distension; it is full of air, and this air cannot find a ready egress during expiration, in consequence of the state of the bronchial tubes; and hence we have the increase of sonoriety. I have not observed this in cases of typhus fever where there was no consolidation of the lung; but, at the same time, it is to be noted, that we have not specially studied this point in fever cases. When the patient draws a deep breath, we frequently find that the râles go on increasing up to the very end of the inspiratory act; and in certain cases, the following curious circumstance is found, and it is one of those which illustrate what I have termed the silent action of the disease—that on applying the stethoscope you will find perhaps but little râle, or a loose rattle in the larger tubes, or perhaps in some of the secondary bronchial tubes,—that is during ordinary breathing. If you contented yourself with such an examination, you might come to the conclusion that there was very little the matter with the patient's lung—slight mucous disease in the lower part of the trachea, or in the very large tubes. But you may be altogether wrong; for, under these circumstances, it frequently happens, that when the patient draws a deep breath, you are startled with the extent and intensity of the râle running to the very end of the respiratory act. It is in this condition that the patient's life is seriously endangered; for if this bronchial disease does not retrocede, but continues to advance, it may happen that the patient will suddenly fall into a condition of asphyxia, tracheal rattle comes on, and he dies a mechanical death—in fact, he dies suffocated. You may leave your patient apparently going on very well, and be summoned to him in the course of a few hours, and find him *in articulo mortis.* This is a common thing, as I mentioned to you before, in patients in whom the bronchial disease has been overlooked or neglected.—*Virginia Medical and Surgical Journal.*

